# Public e-learning opportunities in anesthesia on YouTube

**DOI:** 10.3389/fmed.2024.1429093

**Published:** 2024-09-19

**Authors:** Armin Niklas Flinspach, Jana Merk, Florian Jürgen Raimann, Angelo Ippolito, Linda Vo, Lea Valeska Blum, Stephanie Noone, Mairen Heumine Flinspach, Jasmina Sterz, Vanessa Neef

**Affiliations:** ^1^Department of Anesthesiology, Intensive Care Medicine and Pain Therapy, University Hospital Frankfurt, Goethe University Frankfurt, Frankfurt, Germany; ^2^Department of Anesthesiology, Intensive Care Medicine and Pain Therapy, Sana Clinic Offenbach GmbH, Offenbach, Germany; ^3^Institute for Medical Didactics and Clinical Simulation, Medical Faculty, University Hospital Frankfurt, Goethe University Frankfurt, Frankfurt, Germany; ^4^Department of Trauma, Hand and Reconstructive Surgery, University Hospital Frankfurt, Goethe University Frankfurt, Frankfurt, Germany

**Keywords:** education, teaching, instructional film and video, anesthesia, catheterization

## Abstract

**Background:**

The increasing knowledge in medicine makes continuous education for clinicians necessary more than ever. The range of skills to be covered in anesthesia is constantly growing. How to optimize complex training in practical skills in an increasingly economized environment remains unclear. The extent and suitability to which video platforms assist in learning basic skills in anesthesia has not been investigated yet.

**Methods:**

To identify appropriate videos on YouTube, we conducted a search (May 1st 2023), including common combinations of synonymous terms, and checked up to the 50th result for relevance. Videos initially deemed suitable were archived and evaluated to exclude duplicates. All included videos were subsequently scrutinized for content. For this purpose, a validated checklist to assess procedural and didactic content was used. Data analysis involved assessing interrater reliability, Spearman’s rho test, and linear regression analysis.

**Results:**

We were able to include 222 videos related to 16 basic skills. The low number of videos found on specific skills was striking. The level of fulfillment illustrating a practical skill was repeatedly found <60%. The consistency of the questionnaire was moderate (Fleiss kappa 0.59). Video runtime displayed a significant correlation (*p* < 0.001) with the number of items accomplished on procedural (|*ρ*| = 0.442, *R*^2^ = 0.196) and didactic items (|ρ| = 0.452, *R*^2^ = 0.153). The professional context of the content creators showed no influence.

**Conclusion:**

The quantity of available material on specific basic anesthesiologic skills varied drastically. In addition, the videos available often revealed significant shortcomings, making it challenging to easily assess the quality of the content. The vast majority of evaluated videos did not reflect the intended approach in a scientifically correct manner or were entirely unsuitable for displaying the procedural requirements.

## 1 Introduction

The increasing knowledge in medicine makes continuous education for clinicians necessary more than ever (1). The importance of continuing education has recently grown in academic postgraduate training in the medical field (2). To maintain a high level of education and knowledge, there is a significant need for high-quality, evidence-based content (3). A decision based on a lack of knowledge or an improperly performed procedure may lead to fatal consequences.

Therefore, various forms of protected learning spaces and upstream knowledge have been investigated, particularly in the field of practical skills (4). Instructional videos available on public video platforms are a successful way of upstream education (5–7). The relevance of these tools has increased significantly, especially in times of COVID-19 pandemic, as demonstrated previously (8).

Despite the seemingly inexhaustible availability of educational videos on YouTube, even for specific, complex, and rare procedures, the algorithms developed by YouTube to optimize search results use unclear factors (9–11). As a result, many videos do not correspond to the expertise sought, and the information in video form sometimes proves to be inappropriate and unsuitable (12–14).

There is a distinct conflict between learning skills during residency and commitment to patient’s care during everyday anesthesiologic patient care. Globally, an anesthesiologist must be available at all times to respond to any problems that may arise. The range of skills to be covered is constantly growing (15, 16). In the field of anesthesiology, numerous complex, and not easily transferable procedures are expected in terms of practical skills in clinical practice (17). Generally, the anesthesiologist is considered an airway specialist and therefore needs to be familiar with both simple and complex airway management. Moreover, they need to be specialized in complex vascular approaches and local anesthesia (e.g., peripheral block anesthesia and neuraxial procedures for pain treatment after operations or during childbirth) (18). How this complex and broad training in diverse practical skills can best be achieved in an increasingly economized world remains largely unclear (19, 20).

The extent to which these basic skills of anesthesiological practice with regard to airway management, central line or arterial cannulation, neuraxial procedures, and peripheral block anesthesia are covered on platforms such as YouTube has not been investigated yet. Neither has the suitability of the available video material been investigated to provide assistance in learning these skills by correctly presenting the technical or didactic content in its comprehensive complexity.

The present study aimed to conduct an analysis of anesthesiological basic skills on public available video platforms focused on the availability and quality of videos. For this purpose, a modification and consecutive further developed of a checklist was carried out for the evaluation of the suitability for independent knowledge enhancement.

## 2 Materials and methods

The study was conducted according to the ethical principles of the Helsinki Declaration (Ethical Principles for Medical Research Involving Human Subjects) (21). Since the present study was an evaluation of publicly available videos and not clinical research in humans, the institutional review board (IRB; ethics committee of the University Hospital Frankfurt) decided that no ethical vote was required. This manuscript adheres to the current CONSORT guidelines (22).

### 2.1 Screening and assessment of the videos

To detect suitable published videos on the YouTube platform,^1^ the same search terms were used in both English and German.

The terms were based on the corresponding anesthesiological skill and thus included common combinations of various synonymous terms. A detailed description of the related search terms can be found in Supplementary material I.

The results of the different search terms suggested by the YouTube algorithm were checked regarding their inclusion up to the 50th search result. Hereby a total of 5,200 videos were checked by independent reviewers for their suitability to represent the corresponding skills (the last video access was on May 01, 2023). Videos that were considered suitable based on their title and description were transferred into an Excel sheet via hyperlink. Videos covering lecture courses, duplicates or bare animation videos were excluded from further analyses. Inclusion criteria were a full demonstration of the intended skill on adults in English or German language. For videos meeting the inclusion criteria, the content creator/producer, the date of upload, and duration were recorded.

Eligible videos were grouped into five skill-associated categories that subsumed related basic anesthesiology skills, such as neuraxial procedures (including epidural catheterization and spinal anesthesia) and the categories of arterial and central venous catheterization, peripheral nerve blocks, and airway management. In addition, videos were categorized by content creators. For this purpose, consensus was reached among the reviewers to categorize content creators into one of three groups: medical societies or medical schools, hospitals/hospital chains, and private content creators. This classification has been described previously (9).

### 2.2 Procedural and didactic evaluation of the videos

All videos included in the study were subsequently scrutinized in terms of content. To facilitate this, two checklists to evaluate both procedural and didactic content of educational videos were applied. To evaluate the *didactic approach* of the videos, an already established, evaluated and repeatedly applied checklist was used, which was initially created by Rüsseler et al. (23, 24). The checklist to evaluate the *procedural approach* was developed by the study team consisting of experienced anesthesiology specialists and professionals in medical didactics. Before drafting the procedural checklists, a literature search of international guidelines for corresponding anesthetic procedures was conducted, forming the basis for the items to be evaluated (25–30). The checklist development involved a panel of experts following a previously published process for evaluating relevant items under the moderation of experienced medical didactics experts (9, 24). This was achieved in a consensus phase using a modified three-stage Delphi model. Initially, reviewers were requested by the principal investigator to submit items for checklists regarding the technical completeness of the procedures, which were collected and consolidated into a concerted collection. The experts involved were all experienced specialists in the field of anesthesiology who were familiar with the anesthesiologic procedures tested from regular clinical practice and also had regular teaching experience in these procedures. In a second step, the sum of items determined by the principal investigators was resubmitted to the reviewers, taking into account the valid professional recommendations, with the request to evaluate the individual items with regard to their necessity for correct assessment and necessary illustration in the context of practical training within a video training. The responses were repeatedly collected by the principal investigator and outstanding issues were identified. In a third step, the discrepancies raised by the experts with regard to individual items were discussed in an anonymized video conference and agreed upon with regard to inclusion in the final checklist. This led to the identification and inclusion of 17 items in the checklist, with the degree of fulfilment determined using a modified Likert scale.

The selected process items were then repeatedly tested by a subgroup of reviewers. In order to achieve sufficient test–retest reliability for application to the videos under consideration, the new questionnaire and its items were tested on three selected videos from existing studies.

The procedural checklist addressed aspects of prior preparation of the procedure to be performed, as well as aspects of hygiene, including sterile handling (if appropriate), and correct handling of the equipment for the procedure. The didactic checklist addressed aspects of sound and visual quality, comprehensible terminology, adequate explanations, a learning objective and the reproduction of a comprehensible chronological flow of the procedure (Supplementary material II).

### 2.3 Statistics

Video data were collected using Windows Excel (Office 365, Microsoft Corporation, Redmond, WA, USA). A statistical analysis plan was prepared prior to the present study. This plan aims to analyze the individual results of the reviewers in terms of interrater reliability using a correlation coefficient. In addition to the descriptive analysis of the video content found, the degree of fulfilment determined in the checklists was to be checked for correlations and linear regression analysis using a bivariate model with metadata, in particular the play length, the YouTube “likes” and the creator. Data analysis was performed using SPSS (Ver. 29, IBM Corp., Chicago, IL, USA). Data with continuous scales are presented as median (interquartile range). Data with categorical scales are presented as frequencies and percentages. Interrater reliability was analyzed using a correlation coefficient. Correlations were examined using Spearman’s rho test as well as a Mann–Whitney *U* test. A *p*-value of <0.05 was considered statistically significant.

## 3 Results

**Figure 1:**
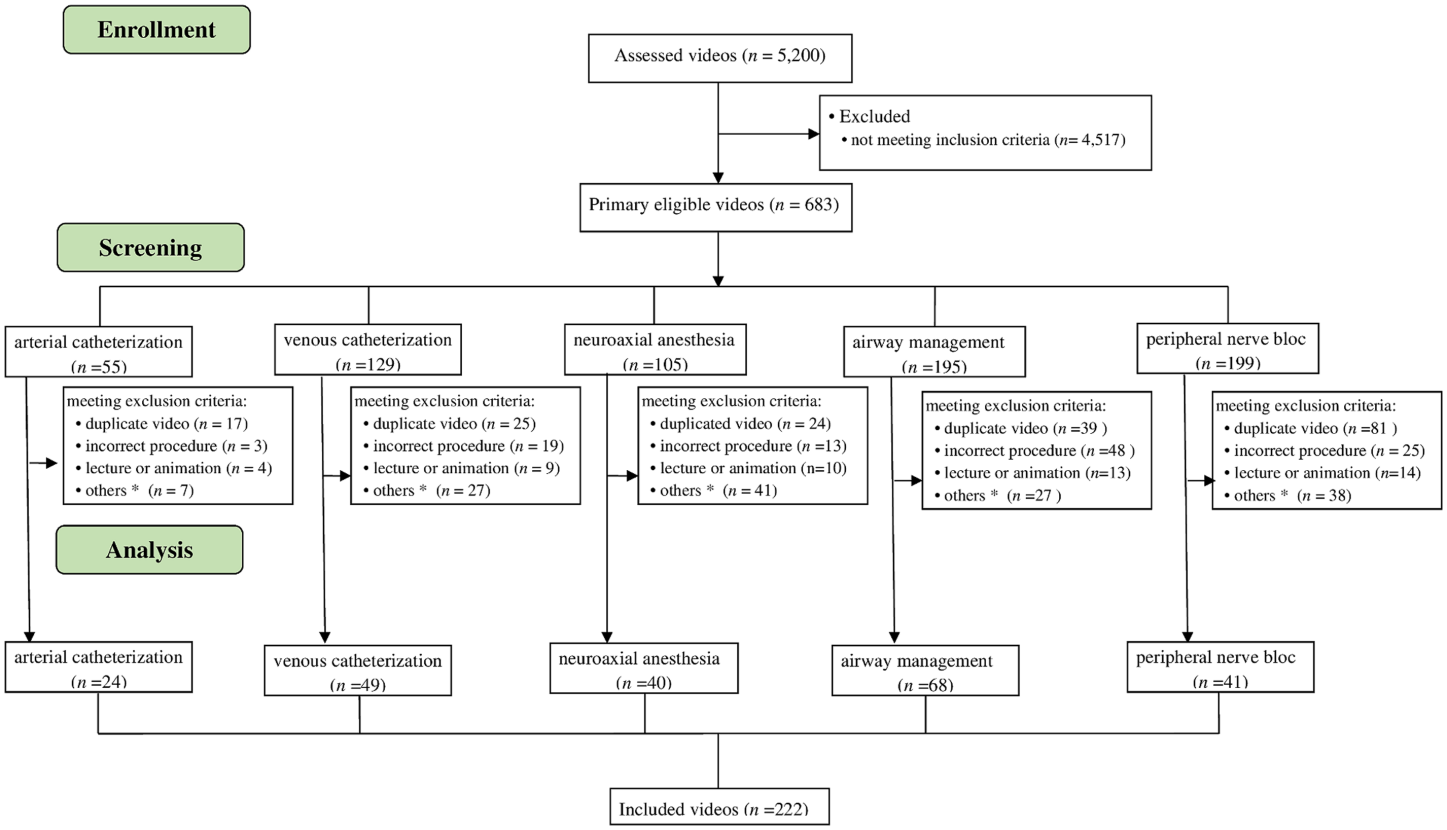
Screening and inclusion process as CONSORT flowchart. The flowchart illustrates the screening and inclusion process of the different skill sets. n, number. *Related skill, incorrect localization, special techniques, self-portrayal.

Of the 5,200 videos screened, 683 were deemed to demonstrate a practical skill. The subsequent screening process based on the exclusion criteria, e.g., duplicates, is illustrated in Figure 1. After eliminating all unsuitable videos, 222 videos related to the 16 basic skills of anesthesiology were included in the study.

### 3.1 Overview of video material

**Figure 2:**
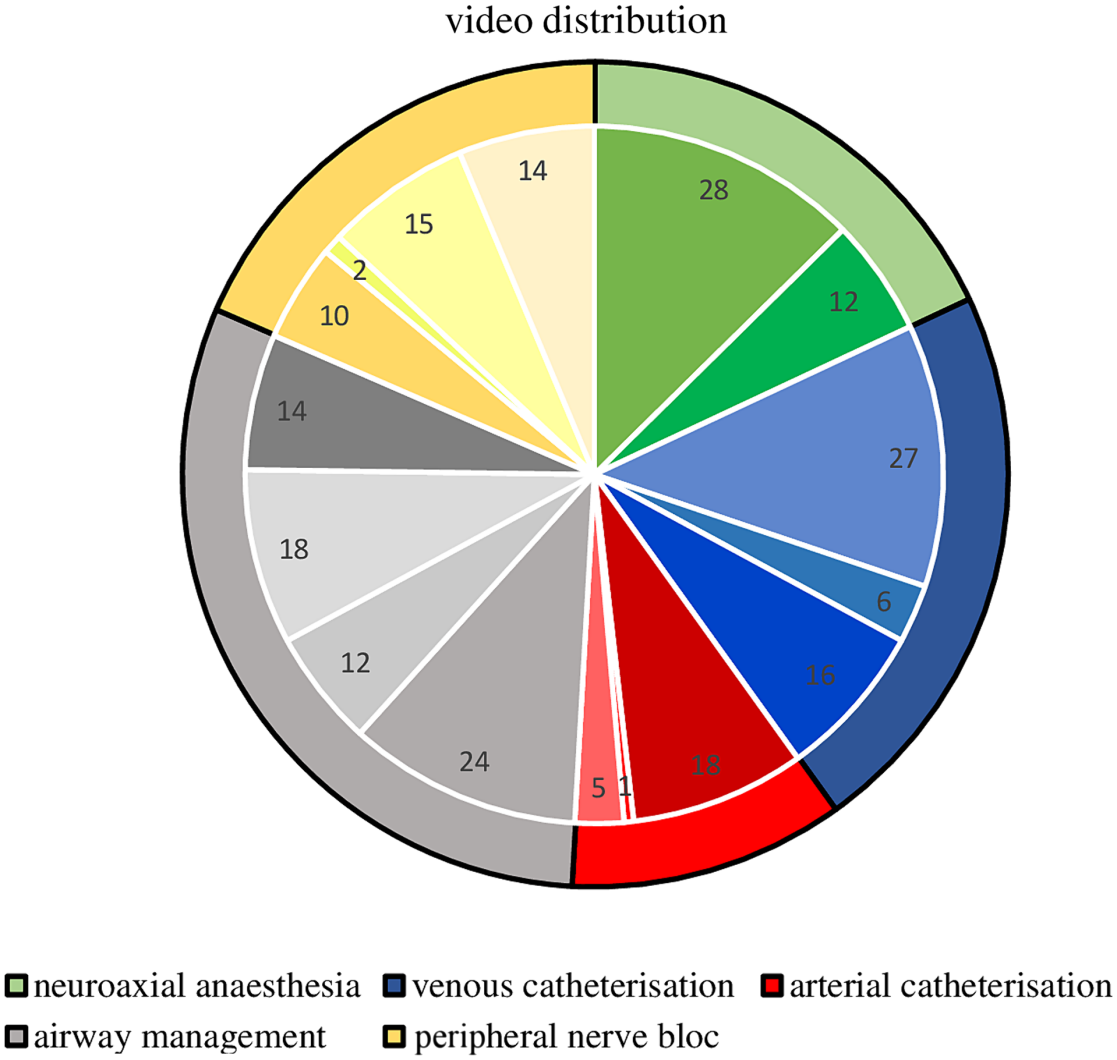
Pie chart of the skills distribution within the 222 analyzed videos. Pie chart graphical illustration of the video material available regarding basic anesthesiology skills, divided into the five selected categories with an internal subdivision into the single skills and total number of videos available regarding those skills.

The low number of videos found on specific skills, for example on cannulation of the brachial artery (one video) or femoral artery (five videos), as well as the femoral vein (six videos) or the procedure of carotid plexus anesthesia (two videos). A detailed presentation of the distribution of the video material can be found in Figure 2 and Table 1.

**Table 1:** Quantity and performance of the practical skill within the selected five skill sets.

Practical skill		Procedural items		Didactic items		Runtime^ǂ^
**Peripheral nerve block**	***n* = 41**					
Nervus ischiadicus block	*n* = 14	33%	(28.9/38.4%)	65%	(61.1/70.1%)	04:55
Nervus femoralis block	*n* = 15	32%	(26.8/38.1%)	69%	(61.9/79.6%)	06:16
Plexus carotis block	*n* = 2	22%	-	54%	-	05:21
Plexus axillaris block	*n* = 10	40%	(32.3/43.3%)	70%	(56.7/76.8%)	07:49
**Airway management**	***n* = 68**					
Video laryngoskopy	*n* = 12	23%	(17.7/39.3%)	60%	(53.5/63.4%)	02:32
Fiberoptic intubation	*n* = 18	41%	(32.6/62.5%)	62%	(51.2/77.1%)	06:36
Double lumen tube	*n* = 14	34%	(23.2/38.8%)	57%	(39.9/60.2%)	05:00
Conventional intubation	*n* = 24	25%	(19.6/69.3%)	67%	(51.6/77.0%)	07:08
**Arterial catheterization**	***n* = 24**					
Femoral artery catheter	*n* = 5	47%	(28.0/53.0%)	56%	(43.3/66.2%)	03:55
Brachial artery catheter	*n* = 1	58%	-	71%	-	03:59
Radial artery catheter	*n* = 18	57%	(42.1/69.3%)	67%	(59.6/81.9%)	05:10
**Venous catheterization**	***n* = 49**					
Femoral vein catheter	*n* = 6	55%	(48.6/63.2%)	69%	(61.0/77.3%)	08:01
Subclavian vein catheter	*n* = 16	58%	(44.0/69.5%)	72%	(60.2/81.6%)	08:33
Jugular vein catheter	*n* = 27	68%	(48.9/75.3%)	76%	(61.4/83.5%)	12:25
**Neuraxial anesthesia**	***n* = 40**					
Epidural catheter	*n* = 28	41%	(32.7/59.5%)	52%	(39.9/62.3%)	14:00
Spinal anesthesia	*n* = 12	49%	(42.9/52.7%)	79%	(62.2/82.5%)	07:20

Tabular overview of the video material found and the item fulfillment levels, results presented as median with interquartile range. ǂ Presentation of the median video duration as minutes: seconds [mm: ss].

### 3.2 Quality of video material

**Figure 3:**
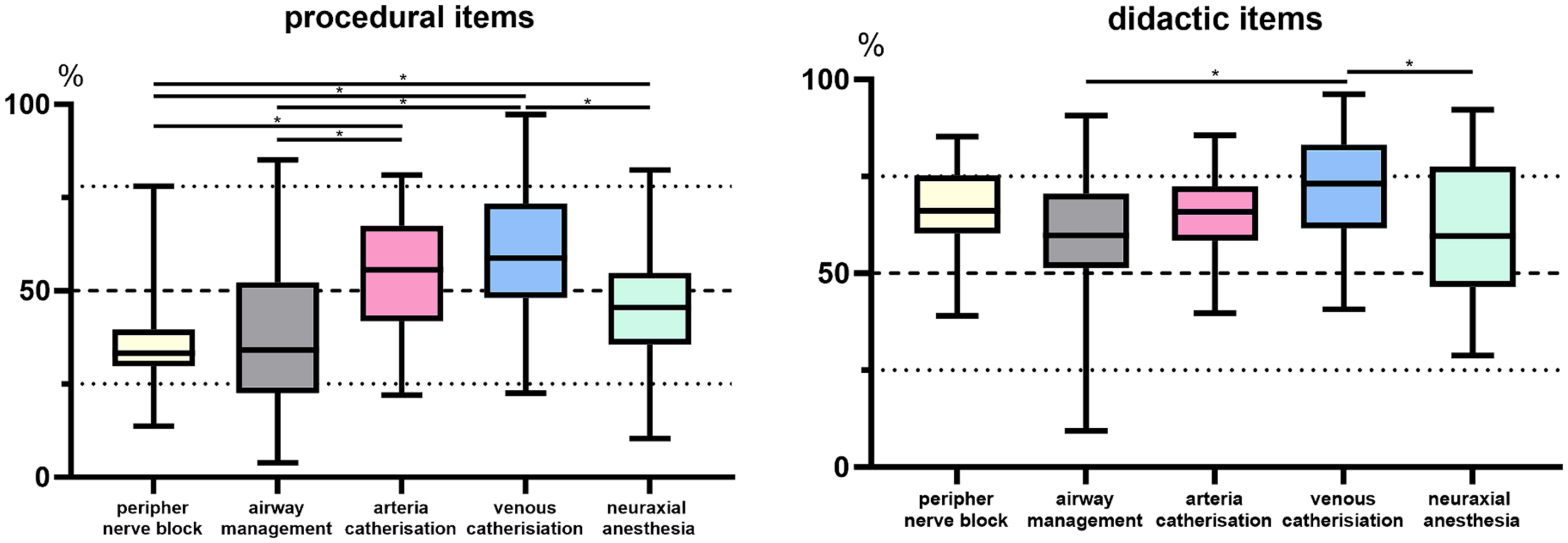
Illustration of the percentage item fulfillment. Boxplot analysis of the percentage fulfillment of the questionnaire-based items subdivided into procedural and didactic aspects and the respective procedural skill group. Bar plot showing significant differences in the degree of fulfillment between the groups *p < 0.05.

The level of fulfillment (measured as percentage of the maximum achievable score) of the presentation of practical skills was repeatedly found to be low, while the didactic items were found significantly more frequently with >60% fulfillment (Figure 3).

Specifically, we found three out of 41 (7.3%) available videos for peripheral nerve blocks with >50% and no video with ≥80% (likely to be suitable for adequate use) fulfillment of procedural items. For airway management, 16 out of 68 (23.5%) videos were found with a score of >50% and two videos with ≥80% fulfilled procedural items. Among the cannulations, arterial 13 (54.1%), venous 36 (73.4%) > 50% item fulfillment were observed, as well as one arterial and five venous cannulation videos with ≥80% item fulfillment. For the neuraxial procedures, 15 videos with >50% and one video with ≥80% were assessed.

The consistency of the procedural questionnaire was rated as moderate to substantial in terms of interrater reliability with a Fleiss kappa of 0.588. The selective assessment of each individual skill was also rated with a Fleiss kappa of uniformly >0.550 (31). The runtime of the videos displayed a significant correlation (*p* < 0.001) with the number of items accomplished on the procedural (|*ρ*| = 0.442) as well as the didactic section (|ρ| = 0.452), but with for the procedural items an *R*^2^ = 0.196 (adjusted *R*^2^ = 0.193) and *R*^2^ = 0.153 (adjusted *R*^2^ = 0.149) indicative as a low goodness-of-fit (32).

Videos varied considerably depending on the shown procedure or the category, e.g., we identified a median run-time of 07:20 min for the placement of a spinal anesthesia within the neuraxial procedures, while the more complex placement of an epidural catheter was demonstrated in videos with a median run-time of 14:00 min, see Table 1 for further details.

The professional context of the content creators, ranging from private users to professional medical service providers up to specialist organizations and government agencies, showed no influence on the successful assessment of items. Likewise, no correlation of the acquired YouTube “likes” to the fulfillment of the checklist items could be proven (|*ρ*| = 0.015).

## 4 Discussion

The present study demonstrates a wide variety in quantity of available material on specific skills on a public video platform such as YouTube. In addition, the examined material often revealed significant shortcomings.

Anesthesiology, being one of the broadest medical fields, encompasses a range of procedures performed routinely on patients. Although videos may serve as a valuable source of learning material, the majority of the videos that were included in the present study did not meet the requirements of an educational video (≥80% of item fulfillment) (33–35). While there has been a trend towards, e.g., didactic improvements observed recently, these are partly due to technical developments, notably in terms of improved video clarity, image stabilization, and sound quality of mobile devices (36, 37). Consequently, it has become relatively easy to film and publish high-quality videos using, e.g., a smartphone (38). This ease of production results in quickly published videos, often lacking prior conception and high-quality content. The evaluated videos cover a spectrum of quality, ranging from self-intubation of the main protagonist to obviously spontaneous and predominantly improvised videos by private individuals to well-conceptualized and reviewed video material from professional associations (watch?v=i82sc11liGk versus watch?v=wDLrRHS7Urw). However, it is extremely difficult for novices to assess the quality of the content presented in response to a search query without a dedicated prior study. Starting the profession itself, rotating to new clinics or departments, presents the novice with the challenge of learning new procedures throughout their career. At this point, the experience to be used as a reference for video evaluation is correspondingly limited. This makes it impossible to judge an instructional video with a procedure-specific trained eye. In this respect, a trained assessor is required to determine a suitable video and provide it to the learner to ensure the correctness and quality of the content.

One aspect of video-associated educational concerns is the learner’s ability to process information in terms of mental load, which, in addition to the structure and didactic quality, is also linked to the runtime of the video (39). However, such relatively short videos are incapable of presenting highly complex processes in an understandable way without overloading the viewer’s capacity for absorption or cutting down on content (40). On the other hand, long videos carry the risk of dwindling attention towards the end of the video. In this context, in the present study the videos on the placement of a jugular vein catheter and the epidural catheterization stand out in particular due to a runtime of more than 12 min. Based on some extremely short videos, we noticed that longer videos were significantly more successful in meeting the requirements in terms of procedural and didactic content, which is particularly evident in complex interventions such as catheterization procedures. The number and high quality of videos available for jugular vein cannulation can be explained by the fact that this approach represents the standard approach for central line placement and therefore targets a wider range of professionals. When analyzing the runtime itself, it is important to bear in mind that individual procedures may differ massively in terms of the number of individual steps to be completed and their complexity. This is exemplified with the neuraxial procedures; a spinal anesthesia (07:20 min) was explained in almost half the median duration, which was used by the creators for an epidural catheterization (14:00 min).

The representation of individual skills was noteworthy, highlighting the fact that skills like arterial cannulation of the brachial artery were only demonstrated in a single video (watch?v=GAq5Di5U4SQ). This is all the more remarkable given that such cannulation is one of the most common access approaches in cardiovascular and vascular surgery for patients with pre-existing cardiovascular disease, together with the femoral artery approach, which was also rarely presented in five videos. Both access routes are essential for modern procedures such as peripheral thermodilution techniques or for alternative approaches in addition to the radial artery in intensive care medicine. While these skills were rarely shown in videos, the frequent presentation of fibre-optic awake intubations, which were found even more frequently than video laryngoscopic procedures, was surprising. In this context, it seems relevant to keep in mind the current discourse regarding the approach to difficult airways that has emerged in recent years due to the increasing and widespread use of video laryngoscopes (41, 42).

The relatively poor performance of the videos on peripheral nerve blocks may be explained by the relatively low level of international harmonization in the technique of these procedures. In the last decade in particular, there has been a rapid advance in these procedures in western industrialized nations from landmark-based punctures to nerve stimulator techniques to ultrasound-guided approaches (watch?v=SpWELgfN1mk and /watch?v=oy_d4YqxRg8). This progress has also been accompanied by a considerable improvement in the hygiene/sterility of the procedure, so that these two factors may have contributed to the results.

Epidural catheter placement is considered one of the most complex of the practical skills observed and has been analyzed several times in terms of its suitability and presence on YouTube with very mixed results (8, 9). It is noteworthy that this procedure, which is considerably more complex to learn than spinal anesthesia, was found more than twice as often (17). This implies that the frequency of video production may be influenced by the subjective or objective degree of difficulty associated with the procedure.

The automatic extension of search results provided by YouTube without a defined end necessitated limiting the search for suitable video material to a set number. However, this introduces the risk of potentially losing relevant video material due to YouTube’s pre-sorting. How YouTube’s algorithm carries out the sorting appears to be unclear; one factor that is repeatedly discussed, originating from other social media, is the number of views and “likes.” (40) However, our research group, along with others, has consistently demonstrated that the number of likes does not correlate with the content or didactic quality of the videos (8, 9, 11, 43, 44). The algorithmic selection of the YouTube algorithm also leads to an extensive pre-exclusion of consistently more than 55% (56–79%) of the displayed content due to the presentation of duplicates or completely incorrect procedures, despite appropriate search terms (45).

Of note, the study has several limitations. First, there is a considerable number of videos not freely available, such of thwarted journals. The creation of digital educational and learning content have become a growing market in recent years, so that in addition to established journals, which publish their content only upon payment or for members, a separate market in the sense of digital apps or encyclopedias with practical handouts including educational videos (e.g., AMBOSS, Berlin) has also emerged. However, the use of this educational content is limited to paying users only. Furthermore, the search algorithms used by YouTube are not disclosed to the public, which could have a decisive influence on which content could be found by the authors with the defined keywords. Regarding the content evaluation, it should be mentioned that although the checklist used was developed in an elaborate process, its items always have the weakness of incomplete representation of procedural and didactic aspects that remain unrecognized (e.g., incorrect sampling or positioning).

The present study focuses on the availability and quality of videos on freely available internet platforms. However, another highly important aspect of this topic covers the utilization and development of checklists to evaluate the quality of the video sources. This should be considered in studies conducted in the future on this topic.

## 5 Conclusion

The video content accessible on the largest public video platform YouTube regarding basic anesthesiologic skills is distinctly limited in terms of individual procedures and suitability for educational purposes. The vast majority of the video material recommended by YouTube’s algorithm does not reflect the intended procedure whatsoever, is duplicated, or is unsuitable for adequately displaying the procedural requirements.

## Data Availability

The original contributions presented in the study are included in the article/Supplementary material, further inquiries can be directed to the corresponding author/s.

## Footnotes

^1^ www.youtube.com
