# Supplementary material for: Public e-learning opportunities in anesthesia on YouTube
Source: Front Med (Lausanne). 2024 Sep 19;11:1429093. doi: 10.3389/fmed.2024.1429093 (PMC11450716; doi:10.3389/fmed.2024.1429093)
Supplement: Supplementary file 1 [file Data_Sheet_1.PDF]

Supplement

Search terms

neuraxial anesthesia

epidural catheter

combinations with “epidural” or “peridural”

and: “anaesthesia” and “birth”	respectively “anesthesia”
“anaesthesia” and “obstetrics”	respectively “anesthesia”
“anaesthesia” and “labour”	respectively “anesthesia”
“catheter” and “birth”	
“catheter” and “obstetrics”	
“catheter” and “labour”	
“catheter” and “caesarean section”	

spinal anesthesia

Video search terms: combinations with “spinal”
and: “anaesthesia” respectively “anesthesia”
“anaesthesia” respectively “anesthesia”
“anaesthesia” respectively “anesthesia”

venous catheterization

jugular vein catheter

Video search terms: combinations with “jugularis” and “catheter”
and:
“venous” or “vein”
or: “central line” German: “Zentral venöser Katheter” OR “ZVK”

femoral vein catheter

Video search terms: combinations with “femoralis” and “catheter”
and:
“venous” or “vein”
or: “central line” German: “Zentral venöser Katheter” OR “ZVK”
or: “femoral catheter”

subclavian vein catheter

Video search terms: combinations with “subclavian” and “catheter”
and:
“venous” or “vein”
or: “central line” German: “Zentral venöser Katheter” OR “ZVK”
or: “subclavian catheter”

arterial catheterization

radial artery catheter
Video search terms: combinations with “radialis”

and “catheter” or “canula”	German: “Katheter“ OR “Katheterisierung”
and: “arteria” or “arterial” or “arterial line”	German: “Ateriell” OR “Arterie” OR “Arterieller Zugang”

brachial artery catheter
Video search terms: combinations with “brachialis”

and “catheter” or “canula”	German: “Katheter“ OR “Katheterisierung”
and: “arteria” or “arterial” or “arterial line”	German: “Ateriell” OR “Arterie” OR “Arterieller Zugang”

femoral artery catheter
Video search terms: combinations with “femoralis”

and “catheter” or “canula”	German: “Katheter“ OR “Katheterisierung”
and: “arteria” or “arterial” or “arterial line”	German: “Ateriell” OR “Arterie” OR “Arterieller Zugang”

airway management

conventional intubation
Video search terms: combinations with “oropharyngeal” and/or “intubation”
and:
“direct laryngoskopy”
“endotracheal”
“endotracheal tube”
“orotracheale tube”

video laryngoskopy
Video search terms: combinations with “oropharyngeal” and/or “intubation”
and:
“video” or “endotracheal”

fiberoptic intubation
Video search terms: combinations with “intubation”
and:

“awake” and “fiberoptic”	German: “wach” OR “fieberoptische”
“awake" and “bronchoskopic”	German: “wach“ OR “bronchoskopische”

double lumen tube
Video search terms: combinations with “intubation”
and:

“Double lumen”	German: „Doppellumen“ OR „Bilumen“
“double lumen tube”	German: “Doppellumentubus“
“Double-lumen endobronchial tube“	

peripheral nerve block

plexus axillaris block

Video search terms: combinations with “block”
and: “axilllary” or “axillaris”
or “brachial plexus” or “plexus brachialis”
or “regional anesthesia/anaesthesia”

plexus carotis block

Video search terms: combinations with “block”
and: “carotid” or “carotis”
or “carotid artery”
or “cervical block”
or “cervical regional anesthesia”

nervus femoralis block

Video search terms: combinations with “block”
and: “femoral” or “femoralis”
or “femoral nerve”
or “regional anesthesia/anaesthesia”

nervus ischiadicus block

Video search terms: combinations with “block”
and: “distal sciatic” or “ischiadicus”
or “distal sciatic nerve”
or “regional anesthesia/anaesthesia”
