# Supplementary material for: Public e-learning opportunities in anesthesia on YouTube
Source: Front Med (Lausanne). 2024 Sep 19;11:1429093. doi: 10.3389/fmed.2024.1429093 (PMC11450716; doi:10.3389/fmed.2024.1429093)
Supplement: Supplementary file 2 [file Data_Sheet_2.PDF]

Inhaltliche Checkliste – Schwerpunkt Inhalt des Videos

Maßnahme	Nicht erwähnt (0 P.)		Falsch/Unvollständig (1 P.)		Richtig (2 P.)
**Initiale Maßnahmen**					
geeignete Materialvorbereitung	☐		☐		☐
Monitoring (EKG, RR)	☐		☐		☐
Anwesenheit einer Assistenz	☐		☐		☐
**aseptisches Arbeiten**					
hygenische Händedesinfektion	☐		☐		☐
Adäquate Handschuhe	☐		☐		☐
Haarnetz, Mundschutz, (steriler Kittel)	☐		☐		☐
Invasive Fertigkeit - steriles Abwaschen	☐		☐		☐
**Airway**[*insofern zutreffen]					
Präoxygenierung	☐		☐		☐
Darstellung & Passage der Stimmbänder	☐		☐		☐
Lagekontrolle des Tubus	☐		☐		☐
Fixierung des Tubus	☐		☐		☐
**Punktion**[*insofern zutreffen]					
Korrekte Lagerung	☐		☐		☐
Punktionsortauswahl	☐		☐		☐
Invasive Fertigkeit - Lokalanästhesie	☐		☐		☐
Nadelführung	☐		☐		☐
Kathetereinbringung	☐		☐		☐
**Weiteres Procedere**					
Anschlussüberwachung	☐		☐		☐
